# Tax effects on foreign direct investment—Just a rerouting

**DOI:** 10.1111/twec.13465

**Published:** 2023-07-10

**Authors:** Dmitry Erokhin

**Affiliations:** ^1^ Vienna University of Economics and Business Vienna Austria

**Keywords:** anti‐tax avoidance rule, bilateral effective average tax rate, indirect FDI, tax haven

## Abstract

This paper investigates tax‐related determinants of indirect foreign direct investment (FDI). In particular, it studies the effects of bilateral effective average tax rates, the strength of anti‐tax avoidance rules in host countries and tax haven status of home countries on the volume of indirect FDI host countries receive. The paper uses the fourth edition of the OECD Benchmark Definition of Foreign Direct Investment (BMD4) database, which distinguishes between ultimate and immediate FDI. Methodologically, the paper relies on the standard gravity equation for FDI and applies the Poisson pseudo‐maximum likelihood estimation model. The paper shows that ultimate FDI is not influenced by tax‐related factors but only real economic determinants, whereas tax rates affect immediate FDI. This finding suggests that previous research may have overestimated the tax elasticity of FDI, and taxes do not have an impact on location decisions of FDI, but rather the route of investing—direct or indirect. The paper defines indirect FDI as the difference between ultimate and immediate FDI and finds that high bilateral effective average tax rates encourage indirect FDI. The finding is robust under different specifications.

## 1 INTRODUCTION

The topic of indirect foreign direct investment (FDI) is receiving increasing attention lately. Governments and citizens are concerned about its possible negative consequences, and numerous media scandals with, for example, leaks of offshore databases or information about non‐payment of taxes by global corporations using conduit structures give the topic even more publicity. What is happening is that pass‐through capital creates a distortive picture of FDI distribution and results in foreign direct investment literally being ‘indirect foreign direct investment’^1^ or ‘foreign (in)direct investment’—‘stateless’ FDI that often even does not ‘touch ground’, also referred to as phantom investment without substance and real links to the local economy (Alabrese & Casella, 2020; Aykut et al., 2017; Borga & Caliandro, 2018; Damgaard et al., 2019; Domínguez‐Jiménez & Poitiers, 2020; Kerner, 2018; Kleinbard, 2013; UNCTAD, 2015; Wamser, 2011).

Indirect FDI undertaken for the reasons of tax and regulation arbitrage rather than productivity and competitiveness considerations may result in tax revenue and welfare losses in home and host countries. Investors route their FDI through third countries to obtain tax benefits not necessarily in the way conceived by host‐country governments. Investors use indirect structures to shift profits from high‐tax to low‐tax jurisdictions to avoid paying taxes. Distorted statistics make it more difficult to understand the role multinational enterprises play in a country, to identify the most important investors in an economy,^2^ to implement sustainable and effective investment policies,^3^ to compare countries in terms of their investment attractiveness and to distinguish long‐term FDI that has a real economic effect and enhances economic growth and purely pass‐through capital. Indirect FDI can be connected with illegal activities such as corruption, tax evasion or money laundering, which endangers regulatory oversight and the rule of law. It can lead to multiple counting^4^ and overestimation of gross FDI (Capolongo et al., 2020). Conduit structures increase inequalities to the detriment of the less technologically developed countries (Pulina & Zanaj, 2021) and could harm competition by allowing multinational companies paying lower effective tax rates than domestic firms (Garcia‐Bernardo et al., 2017; Tørsløv et al., 2022).

Against this background, this paper investigates the reasons behind indirect FDI, where some investors choose to invest through third countries instead of directly. Specifically, it analyses tax‐related determinants of indirect FDI, including bilateral effective average tax rates between host and home countries, the strength of anti‐tax avoidance rules in host countries^5^ and tax haven status of home countries. The paper provides insight into FDI diversion by examining the underlying structures of indirect FDI and presenting a more accurate picture of FDI distribution. Traditional FDI data^6^ cannot always capture the potential rerouting of FDI through third countries. For instance, a high effective corporate tax rate for investors from country *j* in country *i* may not necessarily lead to decreased investment from country *j* in country *i*. Instead, country *j* investors may invest via a third country, such as country *k*, whose investors benefit from preferential treatment in country *i*. As a result, direct FDI from country *j* to country *i* may decrease, but the total FDI from country *j* to country *i* remains unchanged.

The study's results can be summarised as follows: Real economic factors, such as market size and distance, determine ultimate FDI, while the bilateral effective average tax rate negatively impacts immediate FDI. This means that investors alter their investment route when faced with high tax rates, rather than deciding not to invest altogether, indicating a potentially overestimated tax elasticity of FDI in previous research. Tax‐related factors do play a role in the decision to invest indirectly, with higher bilateral effective average tax rates between home and host countries leading to more FDI entering the host countries indirectly, suggesting the presence of tax avoidance strategies in indirect investment structures. Distance is also an essential driver of indirect FDI, with investors preferring conduits that are closer to the host countries, which could be explained by coordination costs, cultural and historical closeness. These findings are robust across various specifications.

The rest of the paper is organised as follows. Section 2 reviews the literature on taxation and FDI. Section 3 introduces the OECD dataset, specifies hypotheses and presents empirical specification. Section 4 summarises and discusses estimation results. Finally, Section 5 concludes.

## 2 LITERATURE REVIEW

The impact of tax rates on FDI has been a subject of much research. One notable meta‐study by Feld and Heckemeyer (2011) found that FDI is highly sensitive to tax rates, with a median tax semi‐elasticity of 2.49 (mean of 3.35) based on analysis of 704 primary estimates.

However, given the structure of traditional FDI data, we only know that immediate FDI declines in response to higher tax rates, but we cannot conclude whether investors facing high‐tax burdens try to circumvent them through some third countries. Indeed, taxes influence the choice of a direct or indirect investing route (Hong, 2018). Mintz and Weichenrieder (2010) were the first to provide empirical evidence for treaty shopping.^7^ Higher bilateral withholding tax rates to (from) Germany increase the probability outward (inward) FDI is rerouted through a third country. Tax motives also play a key role in American multinational companies establishing indirect ownership structures with intermediate owners in third countries (Dyreng et al., 2015; Lewellen & Robinson, 2013).

To overcome the issue of indirect FDI, researchers tend to use firm‐level data. Kalemli‐Özcan et al. (2014) analyse firm‐level data and find that FDI to Europe mostly comes from Europe, but when they go further up the ownership chains, they determine that North American and Asian investors indirectly hold large FDI positions in Europe through different financial centres. Leino and Ali‐Yrkkö (2014) show that inward FDI to Finland largely^8^ consists of pass‐through capital without real economic links to the Finnish economy. Alabrese and Casella (2020) estimate that direct and ultimate shareholders of more than 40% of foreign affiliates worldwide are located in different jurisdictions. However, macro evidence is missing.^9^ Moreover, partly due to a lack of data, most studies on tax avoidance focus on the relationship between conduits and host countries and overlook home countries (Casella, 2019).

The introduction of the OECD BMD4 data brought new insights into the FDI literature. The new OECD dataset gives better results when analysing main FDI drivers (distance, relative country size and trade costs; Fertő & Sass, 2020). It shows huge discrepancies between FDI linkages reported by countries and ‘true’ FDI linkages (Dellis et al., 2020; Saprikina et al., 2020; Szunomár, 2020). Though these differences should not necessarily indicate poor data quality, a systemic analysis of these differences would be helpful to understand financial and tax‐related drivers of firms' decisions and to prepare respective policies (Wacker, 2020).

The new OECD data were also extrapolated to the rest of the world to construct the new more accurate and complete global FDI network by ultimate investors (Casella, 2019; Damgaard & Elkjaer, 2017; Damgaard et al., 2019). Major economies (such as the United States, China [Mainland], the United Kingdom, Germany and France) play a more important role in the new global FDI network. Financial centres (such as the Netherlands, Luxembourg, Hong Kong SAR, Singapore, Ireland and Switzerland) remain key for FDI after the elimination of special purpose entities (SPEs)^10^ (either because of their role in FDI management or because of the difficulty to detect and eliminate SPEs from the data). Phantom investment makes up to 40% of total global FDI. The explanatory power of gravity variables (GDP and distance) increases by roughly 25% when looking at FDI by ultimate investors.

It is all the more important to look behind the FDI structures, given the large role that tax havens play. Tax havens account for at least 30% of global FDI (Haberly & Wójcik, 2015) and 40% of multinational profits (Tørsløv et al., 2022). Investors largely prefer a path going through tax havens for both tax and nontax benefits (Barthel et al., 2010).

The emergence of various opaque FDI structures also brings to the agenda the topic of measures against tax avoidance. Anti‐tax avoidance rules are gaining significance in recent tax treaties and becoming more important than FDI promotion (Blonigen & Davies, 2004). Some studies suggest that the rules are a more significant driver of FDI than tax rates (Haufler & Runkel, 2012). Tax‐planning multinationals are more sensitive to taxes given strong rules against tax planning (Sorbe & Johansson, 2017) and consider anti‐tax avoidance rules in their location decision (Reurink & Garcia‐Bernardo, 2020).

## 3 METHODS AND DATA

### 3.1 OECD dataset

This paper utilises the novel OECD BMD4 dataset, which since September 2014 has collected FDI data from the OECD member states following the fourth edition of the OECD Benchmark Definition of Foreign Direct Investment (OECD, 2021a). BMD4 has introduced a concept of an ultimate investing country, which provides a more accurate structure of FDI (OECD, 2015). BMD4 thus contributes to a better understanding of ‘who is really investing where’, delivers an enhanced empirical basis for informed policy dialogue on attracting FDI (Borga, 2016) and fosters a more profound analysis of the effects of productive FDI (Gurova, 2020). The BMD4 database went online in March 2015 (OECD, 2021a) and today contains data on net inward FDI positions (equity and debt) in 17 OECD countries^11^ by immediate and ultimate investors from all over the world including from a country N to a country N to account for round‐tripping^12^ (OECD, 2021b).^13^ The OECD database constitutes a consistent data source for immediate and ultimate FDI, which is its certain advantage (Wacker, 2020).

**FIGURE 1:**
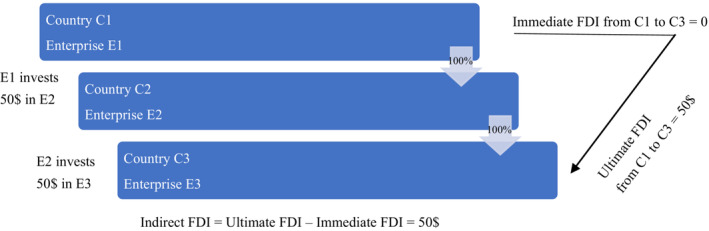
Simple example of immediate investing country and ultimate investing country. [Colour figure can be viewed at wileyonlinelibrary.com]

Figure 1 explains the concepts of immediate and ultimate FDI. Enterprise E1 in country C1 is the ultimate investor in all enterprises below (E2, E3) and the immediate investor for E2. E2 is only the immediate investor for E3 and is used as a conduit by E1 when investing in E3. Traditional FDI statistics shows immediate investor linkages, whereas the new dataset allows figuring out the ultimate investor identified by proceeding up the immediate direct investor's ownership chain until an enterprise is reached not controlled by another entity (more than 50% of the voting power is not owned by another entity; OECD, 2008, 2015). Immediate FDI country is the last step on an investment journey, and ultimate FDI country is where FDI has started its journey.

**FIGURE 2:**
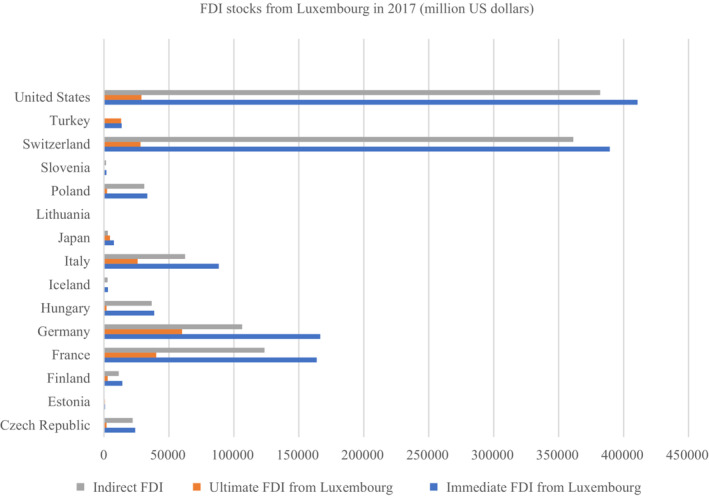
Ultimate and immediate foreign direct investment (FDI) stocks from Luxembourg. [Colour figure can be viewed at wileyonlinelibrary.com]

The differences between ultimate and immediate FDI are indirect FDI. These differences can be tremendous. If we look at immediate and ultimate FDI positions held by Luxembourg in 2017 (Figure 2), in aggregate, only 17% of FDI positions held by Luxembourg were ultimately owned by Luxembourg investors. Given the absolute amount for such a small economy, the number still seems large, which suggests part of this 17% is still not ultimate. As discussed above, financial centres like Luxembourg continue to play a key role for FDI even after the elimination of SPEs from the data, which could be both for the reasons of their real role in FDI management as well as the difficulty to detect and eliminate pass‐through capital from the data (Damgaard & Elkjaer, 2017; Domínguez‐Jiménez & Poitiers, 2020).

The difference between ultimate and immediate FDI positions in country *i* by country *j* in year *t* ($\text{Indirect}\;{\mathrm{FDI}}_{i,j,t}={\mathrm{FDI}}_{i,j,t}^{\text{ultimate}}-{\mathrm{FDI}}_{i,j,t}^{\text{immediate}}$) is defined as indirect FDI. It is the main variable of interest in this paper. According to the OECD BMD4 dataset, ultimate FDI is net inward FDI stock by the ultimate investing country, and immediate FDI is net inward FDI stock by the immediate investing country. The main components of FDI are equity and debt instruments. The use of FDI stocks over FDI flows is preferred because they are less volatile, less subject to endogeneity biases and cover many countries (Enders et al., 2006; Nunnenkamp & Spatz, 2003). Moreover, FDI stocks are found to be good proxies for the activities of multinational companies (Wacker, 2013, 2016). Countries are observed during 2011–2020 (unbalanced sample) (see footnote 19 for the coverage).

### 3.2 Hypotheses

The choice of indirect investment structures may be driven not only by tax optimisation but also by geographical and organisational reasons.^14^ The paper both incorporates the location‐based approach in which investment decisions are taken based on the economic geography of country‐specific characteristics^15^ and analyses tax optimisation as one of the main drivers of indirect FDI. The paper analyses North–North FDI between OECD and European countries where horizontal motives are prevalent (Schneider & Wacker, 2020). Thus, it relies on the standard gravity equation for horizontal FDI (Kleinert & Toubal, 2010) and enriches it with tax‐related factors such as tax rates, strength of anti‐avoidance rules and tax havens. The gravity model is found to perform the best theory‐consistent out‐of‐sample prediction when explaining global FDI patterns (Schneider & Wacker, 2020), and traditional gravity variables have high inclusion probabilities when explaining bilateral FDI (Blonigen & Piger, 2014).

The paper analyses the effect of tax rates, tax havens and anti‐tax avoidance rules on indirect FDI. It assumes that with higher tax rates, investors look for possibilities to reduce the tax burden. One of the potential ways to do so is to redirect FDI to the recipient country via third countries, which have a lower bilateral effective average tax rate with the recipient country. This tax‐driven choice of third countries is called treaty shopping. The classical example of treaty shopping is when a resident of a third state ‘shops’ into a treaty between two other states to gain treaty benefits through a conduit company in a state with a favourable tax treaty (De Broe, 2008). Petkova et al. (2020) show that there are ample possibilities for treaty shopping, which in many cases reduce the actual tax burden^16^ to zero.

Given that most countries in the dataset have a tax treaty with one another, it is not enough to have the presence of a tax treaty as a dummy variable. Moreover, it is not sufficient to include the statutory corporate tax rate only, since there are other factors such as the exemption method or withholding taxes that do matter for foreign investors. Location choice is sensitive to both home and host‐country tax regimes (Mintz & Weichenrieder, 2010). Thus, the paper uses country‐pair‐specific effective tax rates and relies on bilateral effective average tax rates (BEATRs) for inbound investment by Spengel et al. (2020), which are calculated following the Devereux/Griffith methodology and account for corporate tax rates, capital allowances, treatment of inventories, effective real estate tax rates, treatment of foreign intercompany dividends and interests, and withholding tax rates.^17^ BEATRs are superior to the statutory tax rates when measuring the corporate tax burden (Bellak & Leibrecht, 2009) and are crucial for the location decision of an investment (Heinemann et al., 2018; Overesch & Wamser, 2009). Effective tax rates show what investors really pay instead of what they are supposed to pay (Bolwijn et al., 2018).

Hypothesis 1 A higher bilateral effective average tax rate (BEATR) between recipient country *i* and partner country *j* increases indirect FDI from *j* to *i*.

In defining whether a country is a tax haven, the paper follows the tax haven list by Hines (2010). In particular, the interest lies in the so‐called conduit offshore financial centres, which are attractive for routing FDI for tax‐ and nontax‐related reasons (Garcia‐Bernardo et al., 2017; IMF, 2014). Investors may use tax havens inter alia to generate and tax profits with a low tax rate, to exploit tax treaty networks, to reduce governance and measurement transaction costs and to have an access to efficient institutions and foreign stock markets. The expectation is that tax haven benefits make it attractive to invest through tax havens with immediate FDI coming from tax havens being higher than ultimate FDI.

Hypothesis 2 Tax havens^18^ are predominantly used as conduit countries.

In determining the strength of anti‐avoidance rules, the paper follows Johansson et al. (2016).^19^ They assign a score to each OECD and G20 country, which reflects the strength of its anti‐avoidance rules. Strong anti‐tax avoidance rules are predicted to make it less attractive to forward investments via third countries. Consequently, ultimate and immediate FDI converge and indirect FDI decreases.

Hypothesis 3 In response to stronger anti‐tax avoidance rules in the host country *i*, indirect FDI from *j* to *i* decreases.

### 3.3 Estimation methodology

For the empirical analysis, panel data analysis is used. Methodologically, the Poisson pseudo‐maximum likelihood (PPML) estimator by Silva and Tenreyro (2006) is applied,^20^ which is gaining popularity and becoming ‘the workhorse gravity model estimator’ (Shepherd, 2016).

The data allow figuring out which countries invest indirectly and which countries are used as conduits for indirect investments. Following this, the countries are split into two groups: (1) indirect investors and (2) conduit countries, which are both explained below.

**FIGURE 3:**
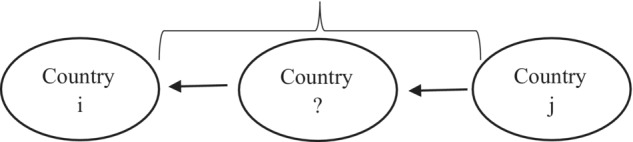
Foreign direct investment (FDI) going indirectly from *j* to *i* through some third countries.
$$\begin{array}{c} \text{Indirect}\;{\mathrm{FDI}}_{i,j,t}=\beta_{0}+\beta_{1}\ln\left({\mathrm{GDP}}_{i,t}\right)+\beta_{2}\ln\left({\mathrm{GDP}}_{j,t}\right)+\beta_{3}\ln\left({\text{DIST}}_{i,j}\right)+\beta_{4}{\text{BEATR}}_{i,j,t}+\beta_{5}{\text{TaxHaven}}_{j}\\ \;+\beta_{6}{\text{AntiAvoidanceRule}}_{i,t}+\alpha_{i}+\gamma_{j}+\lambda_{t}+u_{i,j,t}, \end{array}$$

1. Indirect investors. Here, the paper refers to country‐pair‐year observations, where ultimate FDI from country *j* to country *i* in year *t* outweighs immediate FDI from country *j* to country *i* in year *t* $\left(\text{Indirect}\;{\mathrm{FDI}}_{i,j,t}>0\right)$,^21^ that is country *j* does not only invest directly to country *i* but also channels FDI through some third countries. For example, in 2011, Germany ultimately held an FDI position of 85,929 million US dollars in France, whereas its immediate position was 77,300 million US dollars. This implies that German investors held at least^22^ 8629 million US dollars in France indirectly through some third countries. Figure 3 illustrates schematically what positive $\text{Indirect}\;{\mathrm{FDI}}_{i,j,t}$ implies: country *j* invests to country *i* through some unknown country/countries.

where $\text{Indirect}\;{\mathrm{FDI}}_{i,j,t}$ is indirect FDI from a home country to a host country, $\ln\left({\mathrm{GDP}}_{i,t}\right)$ is the logged GDP of the host country, $\ln\left({\mathrm{GDP}}_{j,t}\right)$ is the logged GDP of the home country, $\ln\left({\text{DIST}}_{i,j}\right)$ is the logged distance between the host and the home country, ${\text{BEATR}}_{i,j,t}$ is the bilateral effective average tax rate between the host and the home country, ${\text{TaxHaven}}_{j}$ is the tax haven status of the home country, ${\text{AntiAvoidanceRule}}_{i,t}$ is the index showing the strictness of anti‐tax avoidance rules in the host country, $\alpha_{i}$ are host‐country fixed effects, $\gamma_{j}$ are home‐country fixed effects, $\lambda_{t}$ are year fixed effects,^23^ and $u_{i,j,t}$ is the error term.

**FIGURE 4:**
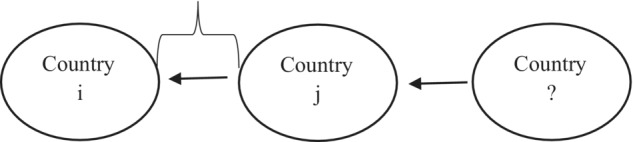
Foreign direct investment (FDI) going indirectly through *j* to *i* from some third countries.
$$\begin{array}{c} \left|\text{Indirect}\;{\mathrm{FDI}}_{i,j,t}\right|=\beta_{0}+\beta_{1}\ln\left({\mathrm{GDP}}_{i,t}\right)+\beta_{2}\ln\left({\mathrm{GDP}}_{j,t}\right)+\beta_{3}\ln\left({\text{DIST}}_{i,j}\right)+\beta_{4}{\text{BEATR}}_{i,j,t}+\beta_{5}{\text{TaxHaven}}_{j}\\ \;+\beta_{6}{\text{AntiAvoidanceRule}}_{i,t}+\alpha_{i}+\gamma_{j}+\lambda_{t}+u_{i,j,t} \end{array}$$

2 Conduit countries (e.g. Luxembourg, see Figure 2). Here, the paper refers to country‐pair‐year observations, where immediate FDI from country *j* to country *i* in year *t* outweighs ultimate FDI from country *j* to country *i* in year *t* $\left(\text{Indirect}\;{\mathrm{FDI}}_{i,j,t}<0\right)$, that is country *j* is used as a conduit country. For example, in 2013, Belgium held an ultimate FDI position of 3102 million US dollars in Italy, whereas its immediate position was 19,309 million US dollars. This means that at least 16,207 million US dollars were channelled through Belgium to Italy by some third countries. Thus, Belgium was used as a conduit for investing in Italy. Figure 4 illustrates schematically what negative $\text{Indirect}\;{\mathrm{FDI}}_{i,j,t}$ implies: some unknown country/countries invest in country *i* indirectly through country *j*.

The absolute value of indirect FDI is used to allow conducting PPML estimation in Stata. This is to be considered when interpreting the estimation results. Otherwise, the variables are the same as in the first case.

## 4 RESULTS AND DISCUSSION

Column 1 Table 1 summarises the results for ultimate FDI.^24^ Host‐country GDP, which is indicative of its market size, has a positive and significant impact on ultimate FDI. It is in line with the size‐of‐market hypothesis that a larger market exhibits economy of scale that contributes to cost reductions and thus attracts foreign investment (Balassa, 1966; Scaperlanda & Mauer, 1969). The same is true for home‐country GDP. Firms from countries with a larger market are more likely to invest abroad (Kyrkilis & Pantelidis, 2003). Large markets allow domestic firms to realise economies of scale, to benefit from country‐specific agglomeration advantages and to develop ownership advantages. With the accumulation of intangible ownership‐specific advantages, firms are more likely to undertake FDI to transfer these advantages through the creation of an internal market abroad. The negative and significant distance coefficient may indicate that costs of operating an affiliate abroad increase with distance and make it less attractive to invest in remote locations (Brenton et al., 1999). Inter alia, they may include personnel costs, communication costs, informational costs, exchange rate risks, language and cultural differences. The BEATR does not have an effect on ultimate FDI. It confirms the finding by Dellis et al. (2020) that taxes do not determine genuine FDI.^25^ The findings of the paper and the literature discussed above suggest that when interested in investing in a country and willing to avoid high tax rates investors may choose indirect investment structures to do so. With this, genuine FDI remains unaffected. It is only the channel of investing which may change. The strength of anti‐tax avoidance rules does not have a significant impact as well, which is intuitively clear, since ultimate FDI is genuine FDI and thus unlikely to be affected by these rules (Barthel et al., 2010). This dispels the fears of some authors that strict rules may have a negative impact on real investment.

**TABLE 1:** Estimation results for the determinants of ultimate, immediate, indirect and conduit foreign direct investment (FDI).

	Ultimate FDI	Immediate FDI	Indirect investors	Conduit countries
$\ln\left({\mathrm{GDP}}_{i,t}\right)$	1.293***	0.552	0.397	0.348
	(0.452)	(0.368)	(0.646)	(0.588)
$\ln\left({\mathrm{GDP}}_{j,t}\right)$	0.796**	0.968*	−0.508	0.427
	(0.322)	(0.529)	(0.351)	(0.829)
$\ln\left({\text{DIST}}_{i,j}\right)$	−0.405***	−0.602***	0.056	−0.325**
	(0.053)	(0.058)	(0.070)	(0.137)
${\text{BEATR}}_{i,j,t}$	0.002	−0.029***	0.028**	0.018
	(0.016)	(0.008)	(0.013)	(0.015)
${\text{AntiAvoidanceRule}}_{i,t}$	−0.031	−0.001	0.079	−0.011
	(0.046)	(0.041)	(0.145)	(0.077)
Constant	−45.458**	−25.888	12.859	−8.078
	(18.578)	(19.630)	(18.238)	(24.161)
Number of observations	3071	3071	1449	1573
Pseudo *R*‐squared	.9151	.9161	.9162	.9271
Home‐country FE	Yes	Yes	Yes	Yes
Host‐country FE	Yes	Yes	Yes	Yes
Year FE	Yes	Yes	Yes	Yes

*Note*: Levels of significance: ***p < .01, **p < .05, *p < .1. Robust standard errors clustered by country pairs in parentheses. Countries are observed during 2011–2020 (unbalanced sample).

Column 2 Table 1 summarises the results for the immediate FDI determinants. Home‐country GDP and distance remain significant, whereas host‐country GDP is not significant anymore, which is in line with the discussed literature that real economic variables have a higher explanation power for the ultimate FDI data, though FDI diversion may still to a certain extent follow the same pattern as ultimate FDI with respect to gravity variables (Weyzig, 2013). The negative and significant tax rate coefficient suggests that higher tax rates discourage investors from investing directly (immediately) so that investors may change the investment route they take but not the decision whether to invest or not to invest in the given location. An increase in the BEATR by one percentage point leads to a 2.90% decrease in immediate FDI, which lies between the median tax semi‐elasticity of 2.49 and the mean semi‐elasticity of 3.35 in the meta‐study by Feld and Heckemeyer (2011).

To overcome negative tax effects, firms look for indirect ways of investing. Column 3 Table 1 summarises the results for the determinants of indirect FDI (these are cases where ultimate FDI is greater than immediate FDI. See the definition of indirect investors in 3.3). The findings confirm Hypothesis 1. The positive and significant BEATR coefficient suggests that a higher bilateral effective average tax rate between a home and a host country leads to more FDI from the home country to the host country on an indirect way. An increase in the BEATR by one percentage point leads to a 2.8% increase in indirect FDI. Combined with the above findings, an increase in the BEATR leads to the rerouting of part of immediate FDI via an indirect route. For example, an increase in the BEATR between Germany and Spain by one percentage point in 2019 would increase indirect FDI from Spain to Germany by approximately 126 million US dollars. Gravity variables are not significant, which suggests that the distance between home and host country as well as the size of the host and home‐country economies do not have an impact on the size of indirect investments. The effect of the anti‐avoidance rules is not significant either.

Column 4 Table 1 summarises the results for the determinants of conduit country FDI (these are cases where ultimate FDI is less than immediate FDI. See the definition of conduit countries in 3.3). The negative and significant distance coefficient suggests that more distant countries are used less as conduits. Theoretically, the result can be explained by the increase in coordination costs. Investors from country *j* would choose a third country which is geographically closer to country *i* as an intermediate to have a more effective coordination (Kalotay, 2012). The BEATR and the anti‐tax avoidance coefficients are not significant.

According to Hypothesis 2, tax havens are expected to be predominantly used as conduit countries. However, home‐country fixed effects absorb tax haven status. Home‐country fixed effects are lifted to conduct the respective regressions. Table 2 summarises the results on the role of tax havens in indirect FDI structures. The tax haven coefficient is positive and significant for all types of FDI. This suggests that the general international financial centres invest more than other countries irrespective of the FDI type. This finding supports the interconnection of offshore investment hubs and the existence of multilayer conduit structures (Bolwijn et al., 2018; Lejour et al., 2021). It confirms the finding by Damgaard and Elkjaer (2017) that financial centres do continue to play an important role even after elimination of indirect FDI structures in the data. This finding could demonstrate that the present data is still not perfect and further work is necessary to detect real FDI networks.

**TABLE 2:** Estimation results for the role of tax havens.

	Ultimate FDI	Immediate FDI	Indirect investors	Conduit countries
$\ln\left({\mathrm{GDP}}_{i,t}\right)$	1.237**	0.509	1.066	0.728
	(0.636)	(0.614)	(0.907)	(0.824)
$\ln\left({\mathrm{GDP}}_{j,t}\right)$	1.055***	0.629***	1.271***	0.183**
	(0.063)	(0.095)	(0.102)	(0.078)
$\ln\left({\text{DIST}}_{i,j}\right)$	−0.412***	−0.557***	−0.124	−0.963***
	(0.044)	(0.084)	(0.138)	(0.118)
${\text{BEATR}}_{i,j,t}$	−0.009	−0.031	0.044*	0.051*
	(0.024)	(0.026)	(0.023)	(0.028)
${\text{AntiAvoidanceRule}}_{i,t}$	−0.022	0.006	0.089	−0.038
	(0.054)	(0.053)	(0.170)	(0.098)
${\text{TaxHaven}}_{j}$	1.361***	1.691***	3.085***	2.613***
	(0.188)	(0.146)	(0.340)	(0.223)
Constant	−51.278***	−16.518	−57.846**	−11.206
	(18.926)	(17.293)	(25.152)	(23.405)
Number of observations	3071	3071	1449	1574
Pseudo *R*‐squared	.8663	.7901	.8510	.8321
Home‐country FE	No	No	No	No
Host‐country FE	Yes	Yes	Yes	Yes
Year FE	Yes	Yes	Yes	Yes

*Note*: Levels of significance: ***p < .01, **p < .05, *p < .1. Robust standard errors clustered by country pairs in parentheses. Countries are observed during 2011–2020 (unbalanced sample).

In FDI regressions, numerous variables have been included in relevant studies with meta‐analyses assigning significant inclusions probabilities to many of them (Blonigen & Piger, 2014). This model selection problem can be addressed with the inclusion of time‐varying home‐and host‐country fixed effects as proposed by Anderson et al. (2016). They absorb GDPs of the home and host country as well as the anti‐avoidance rule. Table 3 summarises the regression results. The BEATR is negative and significant in the immediate FDI regression, and positive and significant in the indirect FDI regression, which confirms the above findings. It is now positive and significant for the conduit countries, which counterintuitively suggests that a higher tax rate between host country and conduit should increase the use of the conduit. However, this result is in line with the recent literature. Lejour et al. (2021) shows that conduit countries have relatively high statutory and effective tax rates. Bulatov (2017) also claims that some of the conduit countries have relatively high corporate tax rates but are still used as conduits, since they also offer nontax‐related benefits like developed financial system and a wide tax treaty network, which compensate for high tax rates. Another potential explanation could be that the choice of a conduit country is predominantly driven by bilateral withholding tax rates, which is one of the BEATR components. This offers possibilities for future research to analyse the exact reasons of choosing particular conduit countries for indirect FDI structures.

**TABLE 3:** Foreign direct investment (FDI) determinants with time‐varying home and host‐country fixed effects.

	Ultimate FDI	Immediate FDI	Indirect investors	Conduit countries
$\ln\left({\text{DIST}}_{i,j}\right)$	−0.403***	−0.586***	0.069	−0.342***
	(0.051)	(0.056)	(0.070)	(0.115)
${\text{BEATR}}_{i,j,t}$	0.004	−0.096***	0.072***	0.127***
	(0.032)	(0.032)	(0.021)	(0.048)
Constant	14.642***	18.958***	8.391***	10.151***
	(1.141)	(1.074)	(0.912)	(1.370)
Number of observations	3047	3047	1389	1546
Pseudo *R*‐squared	.9216	.9267	.9432	.9449
Home country—year FE	Yes	Yes	Yes	Yes
Host country—year FE	Yes	Yes	Yes	Yes

*Note*: Levels of significance: ***p < .01, **p < .05, *p < .1. Robust standard errors clustered by country pairs in parentheses. Countries are observed during 2011–2020 (unbalanced sample).

Above, we introduce strict definitions on indirect investors and conduit countries—indirect FDI greater or smaller than zero. However, there may be measurement errors so that a robustness analysis is conducted with less strict criteria. Countries are defined as indirect investors when indirect FDI is greater than 10 or 100 million US dollars, and as conduit countries when indirect FDI is <−10 or −100 million US dollars. Table 4 summarises the regression results. The BEATR coefficient remains positive and significant in the indirect investors' regression.

**TABLE 4:** Foreign direct investment (FDI) determinants with different thresholds for indirect FDI.

	Indirect investors		Conduit countries	
	$\text{Indirect}\;{\mathrm{FDI}}_{i,j,t}>10$	$\text{Indirect}\;{\mathrm{FDI}}_{i,j,t}>100$	$\text{Indirect}\;{\mathrm{FDI}}_{i,j,t}<-10$	$\text{Indirect}\;{\mathrm{FDI}}_{i,j,t}<-100$
$\ln\left({\mathrm{GDP}}_{i,t}\right)$	0.406	0.408	0.366	0.325
	(0.644)	(0.644)	(0.589)	(0.595)
$\ln\left({\mathrm{GDP}}_{j,t}\right)$	−0.511	−0.504	0.434	0.461
	(0.351)	(0.355)	(0.828)	(0.832)
$\ln\left({\text{DIST}}_{i,j}\right)$	0.058	0.060	−0.329**	−0.325**
	(0.070)	(0.071)	(0.136)	(0.138)
${\text{BEATR}}_{i,j,t}$	0.028**	0.028**	0.018	0.018
	(0.013)	(0.013)	(0.015)	(0.015)
${\text{AntiAvoidanceRule}}_{i,t}$	0.078	0.074	−0.010	−0.011
	(0.144)	(0.146)	(0.077)	(0.077)
Constant	12.676	12.424	−8.747	−8.287
	(18.197)	(18.272)	(24.121)	(24.340)
Number of observations	1276	1031	1351	1043
Pseudo *R*‐squared	.9112	.9031	.9222	.9140
Home‐country FE	Yes	Yes	Yes	Yes
Host‐country FE	Yes	Yes	Yes	Yes
Year FE	Yes	Yes	Yes	Yes

*Note*: Levels of significance: ***p < .01, **p < .05, *p < .1. Robust standard errors clustered by country pairs in parentheses. Countries are observed during 2011–2020 (unbalanced sample).

Given that the main hypothesis is validated if an increase in the BEATR induces an increase in indirect FDI given the value of ultimate FDI, ultimate FDI is included as a control variable. Table 5 summarises the regression outcome. The effect of ultimate FDI on immediate FDI is positive and significant as we would expect it because part of the FDI goes immediately (directly). The effect of the BEATR on immediate FDI remains negative and significant. The effect of GDP of the partner country on indirect FDI is now negative and significant, which could lie in the high correlation between GDP and ultimate FDI (larger countries tend to invest more). Moreover, the distance coefficient is positive and significant in the indirect investors' regression. It suggests that investors tend to choose conduit countries in between when the distance between home and host country increases. The effect of BEATR on indirect FDI remains positive and significant.

**TABLE 5:** Foreign direct investment (FDI) determinants with ultimate FDI as a control variable.

	Immediate FDI	Indirect investors	Conduit countries
$\ln\left({\mathrm{GDP}}_{i,t}\right)$	0.016	−0.656	0.362
	(0.262)	(0.469)	(0.603)
$\ln\left({\mathrm{GDP}}_{j,t}\right)$	0.716	−1.469***	0.435
	(0.479)	(0.385)	(0.816)
$\ln\left({\text{DIST}}_{i,j}\right)$	−0.390***	0.295***	−0.329**
	(0.056)	(0.056)	(0.136)
$\ln\left({\text{UltimateFDI}}_{i,j,t}\right)$	0.475***	0.769***	−0.012
	(0.068)	(0.065)	(0.042)
${\text{BEATR}}_{i,j,t}$	−0.016*	0.026**	0.017
	(0.008)	(0.011)	(0.015)
${\text{AntiAvoidanceRule}}_{i,t}$	−0.015	0.084	−0.009
	(0.039)	(0.091)	(0.076)
Constant	−10.487	59.934***	−8.513
	(16.138)	(17.468)	(24.043)
Number of observations	3071	1449	1573
Pseudo *R*‐squared	.9444	.9479	.9272
Home‐country FE	Yes	Yes	Yes
Host‐country FE	Yes	Yes	Yes
Year FE	Yes	Yes	Yes

*Note*: Levels of significance: ***p < .01, **p < .05, *p < .1. Robust standard errors clustered by country pairs in parentheses. Countries are observed during 2011–2020 (unbalanced sample). Ultimate FDI less than zero is set equal to one to allow logarithmising it.

Table 6 presents the results of the full regressions for ultimate FDI, immediate FDI, indirect investors, and conduit countries including further control variables, in particular, institutional quality, infrastructural development, macroeconomic stability and skill level^26^ as well as contiguity, common language, common religion and presence of a regional trade agreement (RTA). The importance of these variables as factors influencing the attraction of FDI has been confirmed in the literature (Baltagi et al., 2008; Bellak et al., 2009; Boateng et al., 2015; Buchanan et al., 2012; Büthe & Milner, 2008; Ghemawat, 2001; Melitz & Toubal, 2014; Noorbakhsh et al., 2001). Ignoring these factors could lead to an omitted variable bias. However, the regression results show that the above findings do not change and are confirmed also after the inclusion of the control variables. The results are in line with the main findings of the paper. There is no effect of the BEATR on ultimate FDI, whereas the effect of the BEATR on immediate FDI is negative and significant, and on indirect FDI positive and significant.

**TABLE 6:** Foreign direct investment (FDI) determinants with control variables.

	Ultimate FDI	Immediate FDI	Indirect investors	Conduit countries
$\ln\left({\mathrm{GDP}}_{i,t}\right)$	1.328***	1.020***	1.247*	0.767
	(0.360)			
$\ln\left({\mathrm{GDP}}_{j,t}\right)$	0.926**	0.920	−0.266	0.659
	(0.384)	(0.602)	(0.327)	(0.859)
$\ln\left({\text{DIST}}_{i,j}\right)$	−0.265***	−0.526***	0.037	−0.228
	(0.095)	(0.100)	(0.148)	(0.146)
${\text{BEATR}}_{i,j,t}$	0.011	−0.035***	0.041***	0.022
	(0.017)	(0.013)	(0.012)	(0.018)
${\text{AntiAvoidanceRule}}_{i,t}$	−0.087	−0.007	−0.243**	−0.141*
	(0.079)	(0.044)	(0.120)	(0.086)
${\text{Institutional quality}}_{i,t}$	0.336	1.399***	−2.948*	−0.580
	(0.650)	(0.513)	(1.597)	(1.000)
${\text{Infrastructural development}}_{i,t}$	−0.511	−0.817	0.513	−0.213
	(0.658)	(0.530)	(1.647)	(0.977)
${\text{Macroeconomic stability}}_{i,t}$	0.198	−1.221**	0.090	−0.090
	(0.318)	(0.530)	(0.785)	(0.852)
${\text{Skill level}}_{i,t}$	0.084	−0.857*	−0.500	−0.890
	(0.516)	(0.480)	(1.329)	(1.378)
${\text{Contiguity}}_{i,j}$	0.224	−0.148	0.160	0.964***
	(0.213)	(0.201)	(0.422)	(0.251)
${\text{Common language}}_{i,j}$	0.227	0.212	−0.435**	−0.337*
	(0.199)	(0.146)	(0.216)	(0.178)
${\text{Common religion}}_{i,j,t}$	1.559***	1.159***	−1.199	−0.558
	(0.527)	(0.351)	(0.734)	(0.432)
$\text{Regional}\;{\text{trade agreement}}_{i,j,t}$	0.114	0.270	−0.090	0.143
	(0.227)	(0.200)	(0.202)	(0.138)
Constant	−51.845***	−37.707*	−14.467	−24.826
	(17.606)	(20.718)	(20.358)	(28.011)
Number of observations	2465	2465	1199	1240
Pseudo *R*‐squared	.9245	.9217	.9286	.9306
Home‐country FE	Yes	Yes	Yes	Yes
Host‐country FE	Yes	Yes	Yes	Yes
Year FE	Yes	Yes	Yes	Yes
Control variables	Yes	Yes	Yes	Yes

*Note*: Levels of significance: ***p < .01, **p < .05, *p < .1. Robust standard errors clustered by country pairs in parentheses. Countries are observed during 2011–2019 (unbalanced sample). Common religion index data is missing for Lithuania; thus it was excluded from the analysis.

In summary, the paper's findings strongly support the first hypothesis, which suggests that there is a positive relationship between the BEATR and indirect FDI. Additionally, the study offers compelling evidence for the second hypothesis, which proposes that international financial centres are preferred for indirect FDI compared with other countries. However, the third hypothesis on the impact of anti‐tax avoidance regulations on FDI is not supported by the data.

## 5 CONCLUSION

This paper has studied the effects of tax‐related determinants of indirect FDI, in particular, bilateral effective average tax rates between home and host countries, the strength of anti‐tax avoidance rules in host countries, and the tax haven status of home countries. With this, it contributes to a better understanding of the question why some investments come from where they have really originated, and some other investments come from third countries. The answer to this question may yield important policy implications and help to design effective investment and taxation policies.

The estimation results reveal the following main findings. Whereas ultimate FDI is determined by real economic variables such as market size and distance, immediate FDI is negatively affected by the bilateral effective average tax rate. This finding suggests that when facing high tax rates investors change their investing route and not their decision to invest or not. This finding also suggests that previous studies may have overestimated the tax elasticity of FDI.

Tax‐related factors do have a significant impact on the decision to invest indirectly. The higher the bilateral effective average tax rate between home and host country, the more FDI enters the home country indirectly. This finding is robust throughout all distinct specifications.

Distance is another important driver of indirect FDI, which increases the more far away are host and home country. Investors tend to choose conduits in between located close to host countries.

The analysis is limited to the set of the OECD countries,^27^ which distinguish between ultimate and immediate FDI in their reporting. Nevertheless, it delivers interesting insights into indirect investment structures. Another limitation of the paper is that it does not consider the potential endogeneity issues deriving from multilateral resistance terms. The paper assumes that an indirect investment route exists and may be more beneficial depending on the BEATR. However, the analysis could further benefit from calculating the lowest possible BEATR on an indirect route. Future research could also look into the determinants of the conduit country choice by analysing other types of relevant taxes such as bilateral withholding tax rates.

## Data Availability

The data that supports the findings of the paper is openly available in the OECD, WDI, CEPII, WEF data bases as explained in the paper as well as Spengel et al. (2020), Hines (2010), and Johansson et al. (2016).

## Footnotes

^1^
Direct is the degree of control over a foreign affiliate, and indirect is the way the beneficial owner arrives at the control (Kalotay, 2012).

^2^
To understand, which of them make decisions and exercise control, bear risks and receive resulting benefits and which are only used for FDI transmission.

^3^
National strategies for attracting investment must be guided by the real picture of FDI (Casella, 2019). Such a picture goes beyond traditional immediate linkages and looks at the countries where investment ultimately comes from—the countries where decisions are made.

^4^
When FDI from country A goes through a transit country B before reaching its final destination C, it is counted twice by traditional FDI statistics—A–B flow or stock and B–C flow or stock. Almost no studies consider the impact of such conduit structures on the validity of the econometric analysis and their implications (Pohl, 2018).

^5^
Subpart F and other controlled foreign company (CFC) rules in home countries could also place a restriction on indirect FDI. However, this question is beyond the scope of this study, given the low variability in these rules in the countries in the dataset.

^6^
Traditional FDI data assume that international investments are made directly to destinations (Nakamoto & Ikeda, 2021). It counts each flow into and out of the country without distinguishing real and pass‐through investments thus inflating FDI flows and positions (Borga & Caliandro, 2018). The increasing use of holding companies and ownership chains to lower the tax burden without necessarily reallocating inputs or production complicates the use of standard FDI measures (Lipsey, 2007). With this, traditional FDI data cannot reveal the way investors structure their FDI. It cannot distinguish between situations where FDI originates in the Netherlands and where FDI originates in Germany but is routed through the Netherlands (Garcia‐Bernardo et al., 2017).

^7^
The situation where a person who is not a resident of either country a party to a treaty seeks certain benefits under the income tax treaty between the two countries (Grady, 1983). One can also refer to it as multilateralising effect when international agreements with countries A and B may indirectly benefit the ultimate investor from country C (Alabrese & Casella, 2020).

^8^
30% of Finland's inward FDI stock at the end of 2011. Moreover, up to 20% of inward FDI are due to round‐tripping activities by Finish investors.

^9^
One way to identify conduit structures in the macro data is to look at the correlation between inward and outward FDI. If it is high for a country, it may indicate that investors use the country as a conduit (Blanchard & Acalin, 2016; Mintz & Weichenrieder, 2010).

^10^
SPEs are ‘legal entities that have little or no employment, or operations, or physical presence in the jurisdiction in which they are created by their parent enterprises which are typically located in other jurisdictions (economies). They are often used as devices to raise capital or to hold assets and liabilities and usually do not undertake significant production’ (OECD, 2008).

^11^
Austria (2012–2020), Canada (2014–2020), Czech Republic (2013–2020), Estonia (2013–2020), Finland (2013–2020), France (2011–2020), Germany (2013–2020), Hungary (2014–2020), Iceland (2013–2020), Italy (2013–2020), Japan (2017–2020), Lithuania (2015–2020), Poland (2013–2020), Slovenia (2017–2020), Switzerland (2014–2020), Turkey (2016–2020) and the United States (2011–2020).

^12^
Round‐tripping is eliminated from the data.

^13^
See the country list in Tables A1, A2, A3, A4 in Appendix A.

^14^
Sáfrányné Gubik et al. (2020) conduct company interviews to obtain information on the motives of investing indirectly. They find tax optimization, geographical distance, global production chain considerations and the aim to conceal the real origin among the main drivers.

^15^
Given a large geographical distance between home and host country, investors may choose intermediary countries located in between as conduits (Sass et al., 2019).

^16^
Cost of channelling corporate income from one country to another in terms of taxes to be paid. In particular, the tax cost between two countries consists of corporate income taxes to be paid in the host country, a nonresident withholding tax on the income of the subsidiary and corporate income taxes in the home country (Petkova et al., 2020).

^17^
See B.3. Austria: Worked Example for Cross‐Border Investment in Spengel et al. (2020) for the calculation methodology.

^18^
The paper refers to the type of tax havens known as international financial centres, in particular, Switzerland, Cyprus, Ireland, Luxembourg, Malta and the Netherlands.

^19^
You can find a description of these anti‐avoidance rules in Section B3 in Appendix B.

^20^
To implement the PPML estimation, the ppmlhdfe Stata command by Correia et al. (2020) is applied, which is the state‐of‐the‐art command and can estimate even complex gravity equations (Silva & Tenreyro, 2021).

^21^
Though immediate FDI may also include FDI, which is rerouted through *j* to *i* by some country *k* ≠ *j*, it does not change the result so far as ultimate FDI is larger than immediate FDI (it may only influence the magnitude). Indirect FDI is positive and goes through some third countries. If, however, immediate FDI is larger than ultimate FDI, then, the country is used as a conduit. Also, see Section B2 in Appendix B.

^22^
If some of the 77,300 million US dollars was invested on behalf of a country *k*, which used Germany as a conduit, then the indirect FDI from Germany to France would be even higher.

^23^
Given the bilateral nature of the main variable of interest (BEATR) and the rare changes in tax rates between two countries, the estimation does not use country‐pair fixed effects because they may absorb the effect of BEATR on FDI.

^24^
Seventy values out of 2622 are negative for ultimate FDI and 203 for immediate FDI. Setting negative values equal to zero implies that FDI does not provide a net contribution to capital accumulation in a host country because of resources repatriation to a home country (Guerin & Manzocchi, 2009).

^25^
Genuine refers to the relationship between countries *j* and *i*. Ultimate FDI from *j* to *i* could also be used to, for example buy a firm in *k*. Then, it would be conduit equity for country *k*. However, the anti‐avoidance rules affect here only the relationship between *j* and *i*.

^26^
See the Global Competitiveness Report by the World Economic Forum for definitions and measurement methodology.

^27^
Nevertheless, the combined inward FDI of these countries exceeds 23% of global FDI (Turban et al., 2020).

## APPENDIX A SUMMARY STATISTICS AND VARIABLES' DEFINITIONS

**TABLE A1:** Analysed countries.

Home countries	Host countries	Tax havens
Austria, Belgium, Bulgaria, Croatia, Cyprus, Czech Republic, Denmark, Estonia, Finland, France, Germany, Greece, Hungary, Ireland, Italy, Latvia, Lithuania, Luxemburg, Malta, the Netherlands, Poland, Portugal, Romania, Slovakia, Slovenia, Spain, Sweden, UK, North Macedonia, Norway, Switzerland, Turkey, Canada, Japan, USA	Austria, Canada, Czech Republic, Estonia, Finland, France, Germany, Hungary, Italy, Japan, Lithuania, Poland, Slovenia, Switzerland, Turkey, USA	Switzerland, Cyprus, Ireland, Luxembourg, Malta, the Netherlands

**TABLE A2:** Variables' definition and data sources.

Variable	Definition	Source
$\text{Indirect}\;{\mathrm{FDI}}_{i,j,t}$	Difference between ultimate and immediate FDI positions in the reporting country (*i*) by the partner country (*j*) in year *t*	Own calculation based on OECD
${\mathrm{FDI}}_{i,j,t}^{\text{immediate}}$	The last country through which FDI transits before entering host country	OECD
${\mathrm{FDI}}_{i,j,t}^{\text{ultimate}}$	The country, where FDI originates	OECD
${\mathrm{GDP}}_{i,t},{\mathrm{GDP}}_{j,t}$	GDP in current US dollars of a country in year *t*	WDI
${\text{DIST}}_{i,j}$	Geographical distance between the capitals of the reporting (*i*) and the partner (*j*) countries	CEPII
${\text{BEATR}}_{i,j,t}$	Bilateral effective average tax rate between the reporting (*i*) and the partner (*j*) country in year *t* following the Devereux/Griffith methodology	Spengel et al. (2020)
${\text{TaxHaven}}_{j}$	Dummy variable indicating if reporting (*j*) country is a tax haven	Hines (2010)
${\text{AntiAvoidanceRule}}_{i,t}$	Index for the strength of anti‐tax avoidance rules in the reporting country (*i*) in year *t*	Johansson et al. (2016) + own calculations following the authors' methodology

**TABLE A3:** Summary statistics.

Variable	Obs.	Mean	Std. dev.	Min	Max
$\ln\left({\mathrm{GDP}}_{i,t}\right)$	2622	27.058	1.837	23.861	30.696
$\ln\left({\mathrm{GDP}}_{j,t}\right)$	2622	26.683	1.715	23	30.696
$\ln\left({\text{DIST}}_{i,j}\right)$	2622	7.343	0.993	4.088	9.292
${\text{BEATR}}_{i,j,t}$	2622	23.707	7.989	8.1	57.6
${\text{AntiAvoidanceRule}}_{i,t}$	2622	5.305	1.298	3	7
${\text{TaxHaven}}_{j}$	2622	0.192	0.394	0	1
${\text{Infrastructure}}_{i,t}$	2622	0.775	0.093	0.566	0.933
${\text{Institutions}}_{i,t}$	2622	0.655	0.104	0.473	0.886
${\text{MacroeconomicStability}}_{i,t}$	2622	0.805	0.138	0.564	1
${\text{Education}}_{i,t}$	2622	0.762	0.067	0.605	0.896
${\text{Contiguity}}_{i,j}$	2465	0.138	0.345	0	1
${\text{Common language}}_{i,j}$	2465	0.088	0.284	0	1
${\text{Common religion}}_{i,j,t}$	2465	0.269	0.248	0	0.911
$\text{Regional}\;{\text{trade agreement}}_{i,j,t}$	2465	0.791	0.406	0	1
${\mathrm{FDI}}_{i,j,t}^{\text{immediate}}$	2622	21,348.11	64,193.53	−10784.34	619,259
${\mathrm{FDI}}_{i,j,t}^{\text{ultimate}}$	2622	19,504.69	66,642.39	−3385.296	702858.8
$\text{Indirect}\;{\mathrm{FDI}}_{i,j,t}$	2622	−1843.417	40,419.29	−392606.7	600214.3

**TABLE A4:** Correlation matrix.

	$\ln\left({\mathrm{FDI}}_{i,j,t}^{\text{immediate}}\right)$	$\ln\left({\mathrm{FDI}}_{i,j,t}^{\text{ultimate}}\right)$	$\ln\left({\mathrm{GDP}}_{i,t}\right)$	$\ln\left({\mathrm{GDP}}_{j,t}\right)$	$\ln\left({\text{DIST}}_{i,j}\right)$	${\text{AntiAvoidanceRule}}_{i,t}$	${\text{TaxHaven}}_{j}$	${\text{BEATR}}_{i,j,t}$
$\ln\left({\mathrm{FDI}}_{i,j,t}^{\text{immediate}}\right)$	1.0000							
$\ln\left({\mathrm{FDI}}_{i,j,t}^{\text{ultimate}}\right)$	0.8841	1.0000						
$\ln\left({\mathrm{GDP}}_{i,t}\right)$	0.4270	0.3713	1.0000					
$\ln\left({\mathrm{GDP}}_{j,t}\right)$	0.4612	0.6018	0.0690	1.0000				
$\ln\left({\text{DIST}}_{i,j}\right)$	0.0573	0.1201	0.2976	0.3200	1.0000			
${\text{AntiAvoidanceRule}}_{i,t}$	0.2423	0.1673	0.6635	0.0143	0.2834	1.0000		
${\text{TaxHaven}}_{j}$	0.2770	0.1688	0.0297	−0.2658	−0.0022	0.0317	1.0000	
${\text{BEATR}}_{i,j,t}$	0.2989	0.2924	0.8059	0.1457	0.3702	0.5656	−0.0176	1.0000

## APPENDIX B DEFINITIONS

### B1 IMPORTANT DEFINITIONS

- Immediate FDI: FDI from *j* to *i*, directly, where the ultimate owner can be located both in *j* and in some third countries *k*.
- Ultimate FDI: FDI from *j* to *i*, directly or indirectly, where the ultimate owner is located in *j*
- Indirect FDI: Ultimate FDI – Immediate FDI
  - Indirect FDI = FDI rerouted through third countries
  - Indirect investors: Ultimate FDI > Immediate FDI
  - Country *j* uses country *k* to invest in country *i*
- Conduit countries: Ultimate FDI < Immediate FDI
  - Country *j* is used by country *k* to invest in country *i*

### B2 INDIRECT FDI CONCEPT

A: *j* → *k* → *i* (Ultimate indirect)

B: *j* → *i* (Ultimate direct)

C: *h* → *j* → *i* (*j* as conduit)

Ultimate FDI from *j* to *i* = A + B

Immediate FDI from *j* to *i* = B + C

Indirect FDI from *j* to *i* = (A + B) – (B + C) = A − C

The data do not allow to measure A − C, thus indirect FDI is assumed to measure A or C. However, it does not affect, whether country *j* is a conduit country or an indirect investor. If indirect FDI > 0, then A > C and *j* is still an indirect investor. If indirect FDI < 0, then A < C and *j* is a conduit country.

### B3 ANTI‐AVOIDANCE RULES

1. Transfer price rules and documentation requirements. An MNE group invests and sets up a subsidiary abroad, which needs to price its transactions with other parts of the group. It may set artificially low or high prices to shift profit out of the country. For example, Davies et al. (2018) find that large French firms avoid taxes by setting lower intrafirm prices to tax haven affiliates than arm's length prices, which results in tax losses of about 1% of total corporate tax revenues in France. Transfer‐pricing regulations determine that transactions between parts of the same MNE group should not be different if these parts were independent, which makes it more difficult to engage in profit shifting. Theoretically, strict rules should result in a higher tax burden and decreased FDI (Buettner et al., 2018). Adverse effects should increase with a higher tax rate since firms engage more in profit‐shifting activities under a higher host country's tax rate. Empirically, when analysing German MNEs the authors do not find a significant effect of the rules on FDI. The authors conclude that further analysis is necessary to understand whether their finding is generalisable to other countries or only specific to German companies that might find it difficult to engage in transfer pricing.
2. Rules on interest deductibility such as thin capitalisation and interest‐to‐earnings rules to prevent the manipulation of debt location. Thin capitalisation describes a situation where a company is financed with a higher level of debt than equity. Tax rules typically allow deducting interest payable on debt, thus lowering the taxable profit. Intercompany debts can then be used to create interest payments deductible in high‐tax countries and taxable in low‐tax countries. Thin‐capitalisation rules limit interest deductibility for loans from related parties. With this, they should increase the tax burden and depress FDI (Buettner et al., 2012, 2018). These limitations should have strong adverse effects on FDI in high‐tax countries where subsidiaries are incentivised to shift profits abroad via high debt levels. Empirically, the authors confirm that the introduction of thin‐capitalisation rules in high‐tax countries leads to a decrease in FDI. De Mooij and Liu (2021) find that thin‐capitalisation rules raise capital costs and can negatively affect real investment by multinationals with the effect being more pronounced in high‐tax countries. The analysis of the reform of the German thin‐capitalisation rules shows that multinational companies change their capital structure in response to stricter rules and with this indicates that these rules are effective in restricting tax planning (Overesch & Wamser, 2010; Wamser, 2014; Weichenrieder & Windischbauer, 2008). Buettner et al. (2012) find an impact of thin‐capitalisation rules on the capital structure of foreign subsidiaries in the OECD countries, which also supports their effectiveness in curbing tax‐planning activities.
3. Controlled foreign company (CFC) rules. A domestic taxpayer controls a company in a low‐or‐no‐tax jurisdiction and shifts passive income from, for example, patents, trademarks and interest to it. CFC rules prescribe a minimum tax rate and provide that the domestic taxpayer pay tax to the residence country at the residence country's tax rate on the passive income earned in a jurisdiction with a below‐the‐minimum tax rate (Dharmapala, 2020). Theoretically, Weichenrieder (1996) shows that CFC rules can lower the cost of capital for real direct investment. Prettl (2017) provides empirical evidence that CFC rules do tend to increase real FDI and decrease passive income in foreign subsidiaries. Empirical evidence also suggests that less stringent CFC rules facilitate profit‐shifting activities (Schenkelberg, 2020).
4. General anti‐avoidance rules (GAARs). Companies may conduct transactions that lack economic substance and have taxable income reduction as the main purpose. GAARs look at the substance of these transactions and can limit or deny tax benefits when transactions do not have a reasonable commercial purpose or where their purpose is to change tax incidence. Illustrative is the Indian example discussed in the Introduction with a significant decrease in FDI flows from Mauritius to India under the fear of the GAAR impact as one of the potential reasons. The same is true for China where the GAAR introduction has led to a reduction in tax avoidance (Leung et al., 2019).
5. Withholding taxes on interest payments, royalties and dividends, taking into account bilateral tax treaties. Withholding taxes are levied on interest, royalties and dividends destined to nonresident entities. They are also analysed in the category of anti‐avoidance rules because of their influence on cross‐border tax‐planning opportunities. For example, they can reduce gains from manipulation of debt location or gains from strategic allocation of intangible assets (Johansson et al., 2016). Białek‐Jaworska and Klapkiv (2021) find that withholding tax on interests reduces international profit shifting by FDI.
